# The Shortage of Husbands

**Published:** 1918-02-23

**Authors:** William H. Walker

**Affiliations:** Officer, Mental Deficiency Act, Guildhall, Hull.


### THE EDITOR'S LETTER-BOX.

### The Shortage of Husbands.

*To the Editor of* The Hospital.

Sir,—I notice in one of your contemporaries a remark on the above by Lady Nott Bower, in which she stated that the shortage of healthy, promising husbands was a thing we could not overlook, and that she was afraid many a girl would be willing to accept the hand of a feeble-minded man whom she would not have looked at before the war.

I am afraid this is too true. Persons like myself, who move about amongst the mentally defective, find there is a great risk of this undesirable class-mating, and consequently reproducing their kind. Experience and statistics prove these people are usually more prolific than normal persons, and in nearly every instance the progeny are as defective or more so than their parents. How necessary it is that they should be discouraged from marrying! Degenerate stocks are tending to increase, and it is the least fit stocks from which the future generation may be recruited. The fact that defectives are earning money to-day is the cause of their needing more care.

Parliament has set apart the sum of £150,000 to be spent in carrying out the provisions of the Mental Deficiency Act of 1913, and it is stated that only £60,000 of that sum has been spent, which I should like to point out does not prove that local authorities have been lax or defaulting in carrying out the Act, but that owing to the war happening just about the time the Act was put into operation, and the shortage of institutional accommodation, with the Government's injunction to economise, they were unable to do as much as they would have done had conditions been normal.—Yours,

William H. Walker,
Officer, Mental Deficiency Act,
Guildhall, Hull.

